# Incidence and risk factors of postdural puncture headache: prospective cohort study design

**DOI:** 10.1186/s13741-020-00164-2

**Published:** 2020-11-09

**Authors:** Bedilu Girma Weji, Mohammed Suleiman Obsa, Kidest Getu Melese, Gedion Asnake Azeze

**Affiliations:** 1 Department of Anesthesia, Saint Paul’s Hospital Millennium Medical College, Addis Ababa, Ethiopia; 2 Department of Anesthesia, Wolaita Sodo University, Wolaita Sodo, Ethiopia; 3 Department of Midwifery, Wolaita Sodo University, Wolaita Sodo, Ethiopia

**Keywords:** Spinal needle, Lumbar puncture, Pencil-point spinal needle, Postdural puncture headache, Spinal anesthesia

## Abstract

**Background:**

Postdural puncture headache is one of the complications following spinal anesthesia and accidental dural puncture. Several modifiable risk factors contribute to the development of headache after lumbar puncture, which includes needle size, needle design, direction of the bevel, and number of lumbar puncture attempts. This study aimed to assess the incidence and risk of postdural puncture headache.

**Methods:**

This prospective cohort study design was conducted using a consecutive sampling method. Regular supervision and follow-up were performed. Data were entered into Epi info version 7 software and transported to SPSS version 20 for analysis. The odds ratio and 95% confidence interval were computed. The findings of the study were reported using tables, figures, and narrations. Variables that were found to be candidates (*p* value < 0.25) on binary logistic regression entered into a multiple logistic regression analysis to identify independent predictors of postdural puncture headache.

**Results:**

One hundred fifty eligible study participants were included in our study, of which 28.7% had developed postdural puncture headache. This study found that needle size, number of cerebrospinal fluid drops, and multiple attempts were significant independent predictors of postdural puncture headache (*p* < 0.05). In addition, twenty-five needles were identified as the strongest preoperative independent predictor of postdural puncture headache (AOR = 4.150, CI = 1.433–12.021)

**Conclusions:**

A recent study revealed that a small spinal needle was much better than a large cutting spinal needle regarding the frequency of postdural puncture headache. In addition, frequent attempts during lumbar puncture and increased cerebrospinal fluid leakage were associated with the events. In view of this, we recommend the use of a small spinal needle to avoid more leakage of cerebrospinal fluid and multiple attempts at spinal anesthesia and lumbar puncture.

## Background

According to the International Classification of Headache Disorder, postdural puncture headache (PDPH) is defined as a headache occurring within 5 days after lumbar puncture (LP), which is aggravated when standing or sitting and relieved when lying flat (Arnold 2018). Associated symptoms include stiff neck, hearing loss, tinnitus, photophobia, hyperacusia, and nausea. The prevalence of PDPH is higher in pregnant women (Chuanjie Wu et al. 2016). It is a common complication of lumbar puncture, which is likely due to the loss of cerebrospinal fluid (CSF) into the epidural space through the dural tear. The reported incidence of PDPH varies from 10 to 40% depending on age, gender, and needle size (Oedit et al. 2005; Kiki et al. 2009; Kwak 2017).

Several factors contribute to the development of headache after lumbar puncture, including needle size, needle design, direction of the bevel, and number of LP attempts. Namely, using smaller diameter and non-cutting (atraumatic) needles is correlated with a lower incidence of headache after LP. Additionally, insertion of the needle with the bevel parallel to the dural fibers facilitates closure of the hole and minimizes cerebrospinal fluid leakage (Ahmed et al. 2006; Seza et al. 2008). Modifiable risk factors of PDPH include needle size, needle shape, bevel orientation and insertion angle, stylet replacement, and operator experience (Bezov et al. 2010a). Needle size might be the most significant factor in the development of PDPH (Flaatten et al. 1989).

Spinal needles generally used today are 22 to 27  G, but sizes ranging from 18 to 30  G are available. The incidence of PDPH after spinal anesthesia performed with Quincke, cutting needle, is 36% with 22 -G needle, 25% with 25- G needle, 2 to 12% with 26- G needle, and less than 2% for smaller than 26- G needles (Turnbull and Shepherd 2003; Lynch et al. 1994). The smaller needle diameter reduces the incidence of PDPH (Castrillo et al. 2015). However, spinal needles, which are extremely thin, can cause technical difficulties for the operator. On the other hand, multiple dural punctures caused by unsuccessful puncture would increase the rate of PDPH (Castrillo et al. 2015; Zhang et al. 2016). Needle design variables, such as the needle size and needle shape, have been modified to enable rapid flow of cerebral spinal fluid (CSF) and injected medications, yet simultaneously limit dural trauma and loss of CSF (Xu et al. 2017). The goal of this study was to determine the incidence and risk factors of PDPH.

## Materials and methods

Prospective cohort study design employed at Wolaita Sodo University from January 2017 to March 2017. All pregnant mothers who gave births by cesarean section were the source population, and all pregnant mothers who gave births by cesarean section under spinal anesthesia at Wolaita Sodo University Teaching referral hospital were the study population. Non-cooperative patients and patients with impaired cognitive ability were excluded from the study. A consecutive sampling technique was used to include study participants.

Structured checklists and questionnaires were prepared in English, which included sociodemographic and perioperative data, the severity of pain, and the duration of analgesia and total analgesia consumption. On arrival of the patient to the operating room, all standard monitors were attached, and baseline heart rate [HR], mean arterial pressure (MAP), and oxygen saturation [SpO_2_] were recorded. The intravenous line (IV) was secured with an appropriate size cannula, and all patients were preloaded with 15 mL/kg of Ringer’s lactate IV before spinal anesthesia. A lumbar puncture was performed at the L2-3, L3-4, or L4-5 intervertebral space with the patient in the sitting or lateral position. All patients received a standard spinal anesthetic consisting of bupivacaine 12–14 mg. A T4-6 sensory dermatome level was obtained before surgical incision. Maternal age, height, weight, parity, and history of previous PDPH were noted, as well as the type of needle and the operator training level. Postoperatively, all patients were seen daily by anesthesia unaware of the type of needle used and were questioned for the presence of a headache.

In this study, PDPH was defined as any headache following spinal anesthesia that develops within 3 days after dural puncture and worsens within 15 min after sitting or standing and improves within 15 min after lying down and is also one of the following associated factors present: neck stiffness, tinnitus, hyperacusia, photophobia, or nausea. When a patient complained of an occipital or frontal headache, she was monitored daily until she was discharged from the hospital. All patients received a telephone call 1 week later to evaluate for any signs or symptoms of a delayed-onset headache. Patients with a headache were evaluated and given standard treatment for the duration of the headache.

Data collectors were trained, and a pretest was performed on 10% of the sample patients. During data collection, regular supervision and follow-up were performed appropriately. Each data point was cross-checked for completeness and consistency every day.

Statistical data analysis was performed using SPSS software version 20. Data were summarized in the form of proportions and frequency tables for categorical variables. In the binary logistic regression analysis, the odds ratio together with the 95% confidence interval was calculated to test for the association between the possible predictors and outcome variables. A *p* value of less than 0.25 was considered a potential candidate for the final model and entered into a multiple logistic regression to determine independent predictors of PDPH. All variables that were found to be statistically significant (*p* value < 0.05) on multiple logistic regression analysis were considered independent predictors of postdural puncture headache.

The primary outcome of this study was the incidence and risk factors of postdural puncture. The secondary outcomes measured were the severity and duration of PDPH.

### Ethical approval and consent

Ethical clearance and approval were obtained from the ethical review committee of the College of Health Science, and official letters were obtained from the anesthesia department of Wolaita Sodo University. Informed written consent was obtained from the study participants. Confidentiality and anonymity of information were ensured.

## Result

### Sociodemographic and preoperative characteristics

A total of 150 pregnant mothers were enrolled in the study, 43 (28.7%) of whom developed postdural puncture headache. The majority of respondents 80 (53.3%) were between the ages of 20 and 25 years. The mean age of respondents was 26.44 ± SD (4.0) (minimum 18 and maximum 40), and the mean BMI of respondents was 22.1 ± SD (2.4) (minimum 18 and maximum 28). Almost all of the respondents 144 (96%) were found in ASA class I, while 6 (4%) of all respondents were found in ASA class II. Regarding previous history of anesthesia, 79.3% of all respondents had no previous history of anesthesia (Table 1).

**Table 1:** Sociodemographic and preoperative characteristics of patients who underwent spinal anesthesia, 2017

Variables	Category	Frequency	Percentage
**Age in years**	18–24	34	22.7
	25–29	80	53.3
	30–34	30	20.0
	35–39	6	4.0
**Parity**	Primigravida	53	35.3
	Multigravida	97	64.7
**ASA class**	Class I	145	96.7
	Class II	5	3.3
**BMI**	Underweight	8	5.3
	Normal	124	82.7
	Overweight	18	12.0
**Previous history of anesthesia**	No	119	79.3
	Yes	31	20.7

### Intraoperative characteristics of respondents

Approximately 52.67% of all respondents took spinal anesthesia by a large gauge needle, while 18% of spinal anesthesia was given by using a medium needle. With respect to the position of the patients during spinal anesthesia administration, almost all of the patients took spinal anesthesia in the sitting position. The majority of spinal anesthesia was administered after three drops and two attempts (Table 2).

**Table 2:** Intraoperative characteristics of patients who underwent spinal anesthesia, 2017

Variables	Category	Frequency	Percentage
**Needle size**	18	39	26.0
	21	40	26.7
	22	14	9.3
	24	13	8.7
	25	44	29.3
**Position**	Sitting position	141	94.0
	Lateral position	9	6.0
**Number of drop**	Less than or equal to three	68	45.3
	Greater than three	82	54.7
**Number of attempts**	One	50	33.3
	Two	69	46.0
	Greater than two	31	20.7

### Independent predictors of PDPH

Risk factors associated with PDPH were analyzed further. All variables that turned out with a *p* value < 0.25 after binary logistic regression analysis were used to model for final analysis. Consequently, backward multiple logistic regression analysis was used to determine the association of PDPH. Accordingly, the output of multiple logistic regression models revealed that needle size, number of CSF drops, and multiple attempts were significantly independent predictors of PDPH (*p* < 0.05). Maternal parity and BMI were not significantly associated with PDPH (*p* < 0.05). Twenty-five needles were identified as the strongest preoperative independent predictor of PDPH (AOR = 4.150, CI = 1.433–12.021) (Table 3).

**Table 3:** Factors associated with postdural puncture headache at among patient who underwent spinal anesthesia, 2017

Variable	Category	PDPH		Sig.	Exp(B)	95% CI for EXP(B)	
		No	Yes			Lower	Upper
**Maternal parity**	Primigravida	34	19				
	Multigravida	73	24	.420	1.412	.611	3.265
**Needle size**	18	19	20	.009			
	21	32	8	.009	4.150	1.433	12.021
	22	11	3	.495	.639	.177	2.313
	24	9	4	.722	.740	.141	3.877
	25	36	8	.590	1.498	.344	6.516
**Number of attempts**	1	38	12	.048			
	2	53	16	.131	.443	.154	1.274
	> 2	16	15	.033	.336	.123	.914
**BMI**	Underweight	7	1	.596			
	Normal	89	35	.315	.278	.023	3.385
	Overweight	11	7	.757	.820	.235	2.867
**Number of CSF drops**	1	10	4	.142			
	2	49	15	.021	.180	.021	1.574
	3	43	16	.025	.138	.025	.779
	4	5	8	.024	.135	.024	.766

## Discussion

Needle size is the most important reason in the development of PDPH. The smaller needle diameter has been thought to be effective in reducing the incidence of PDPH (Bezov et al. 2010a; Bezov et al. 2010b). However, very thin spinal needles can cause technical difficulties for the operator, resulting in multiple dural punctures and a high incidence rate of PDPH (Zhang et al. 2016). Spinal needle size, spinal needle shape, multiple attempts, and amount of CSF drops during lumbar puncture are modifiable risk factors of PDPH.

The results of this study indicated that a small pencil point spinal needle has a lower risk of postdural puncture headache, which agrees with other similar studies (Xu et al. 2017; Pal et al. 2011). However, another study conducted to compare 24-gauge Sprotte and 25-gauge Quincke needles and the effect of subarachnoid administration of fentanyl found no significant difference in PDPH between the pencil-point Sprotte and Quincke needle inserted parallel to the dural fibers in obstetric patients (Devcic et al. 1993). However, our study was performed on five different sizes of spinal needles. A few studies demonstrated that Quincke needles, if introduced with the bevel parallel to the longitudinal axis of the dural fibers, could reduce the incidence of PDPH (Flaatten et al. 1989; Corbey et al. 1997).

According to the findings of this study, the incidence of postdural puncture headache was 28.7%, which is lower than the study conducted at Gondar University Hospital, which showed that 38.8% of PDPH cases occurred (Kassa et al. 2015). In contrast to this study, another study conducted on factors associated with the onset and persistence of post-lumbar puncture headache in New York showed that the incidence of PDPH is 21.6% during the immediate post procedural period (Monserrate et al. 2015). This difference may be because they divided the occurrence of PDPH into three periods. The present study showed the total occurrences of PDPH.

This study found that a higher drop in CSF was strongly associated with PDPH, which agrees with other similar studies (Monserrate et al. 2015). This may be due to an acute decrease in intracranial CSF pressure owing to extraction of larger volumes of CSF, triggering meningeal vasodilatation and positional traction on intracranial structures. If no further leakage of CSF occurs following the procedure, the amount of CSF removed should be replenished within several hours at physiological rates of CSF production (Brown et al. 2004).

The findings of this study revealed that multiple attempts during spinal anesthesia administration were significantly associated with PDPH. Another study also found that persistent failure of the dural puncture causes continuous leakage of CSF and contributes to postdural puncture headache. One potential mechanism for the lower rates of PDPH and therapeutic blood patch seen with extraction of higher volumes of CSF may be that transiently lower CSF pressures immediately following the procedure decrease continued leakage through the dural puncture, facilitating dural closure (Sakurai et al. 2013). However, another study found that there is no significant difference observed in the incidence of PDPH between single shot block and two or more attempts (Salik et al. 2018).

## Conclusion

The findings of this study revealed that a small spinal needle is significantly superior to a large spinal needle regarding the occurrence of PDPH. In addition, multiple attempts and the amount of CSF drops were significantly associated with PDPH. In view of this, we recommend the use of a small spinal needle, to avoid multiple attempts and frequent CSF drops during spinal anesthesia and lumbar puncture.

## Data Availability

Available up on request.
